# Reliability of pediatric ventricular function analysis by short-axis “single-cycle-stack-advance” single-shot compressed-sensing cines in minimal breath-hold time

**DOI:** 10.1007/s00330-021-08335-5

**Published:** 2021-10-29

**Authors:** Suzan Hatipoglu, Peter Gatehouse, Sylvia Krupickova, Winston Banya, Piers Daubeney, Batool Almogheer, Cemil Izgi, Peter Weale, Carmel Hayes, David Firmin, Dudley J. Pennell

**Affiliations:** 1 Cardiovascular Magnetic Resonance Unit, Royal Brompton & Harefield NHS Foundation Trust, London, UK; 2 Research Office, Royal Brompton & Harefield NHS Foundation Trust, London, UK; 3 Pediatric Cardiology Department, Royal Brompton & Harefield NHS Foundation Trust, London, UK; 4 Siemens Healthcare Plc, Frimley, UK; 5 Siemens Healthcare GmbH, Erlangen, Germany; 6 National Heart & Lung Institute, Imperial College, London, UK

**Keywords:** Pediatric, Diagnostic imaging, Multiparametric magnetic resonance imaging, Ventricular function

## Abstract

**Objectives:**

Cardiovascular magnetic resonance (CMR) cine imaging by compressed sensing (CS) is promising for patients unable to tolerate long breath-holding. However, the need for a steady-state free-precession (SSFP) preparation cardiac cycle for each slice extends the breath-hold duration (e.g. for 10 slices, 20 cardiac cycles) to an impractical length. We investigated a method reducing breath-hold duration by half and assessed its reliability for biventricular volume analysis in a pediatric population.

**Methods:**

Fifty-five consecutive pediatric patients (median age 12 years, range 7–17) referred for assessment of congenital heart disease or cardiomyopathy were included. Conventional multiple breath-hold SSFP short-axis (SAX) stack cines served as the reference. Real-time CS SSFP cines were applied without the steady-state preparation cycle preceding each SAX cine slice, accepting the limitation of omitting late diastole. The total acquisition time was 1 RR interval/slice. Volumetric analysis was performed for conventional and “single-cycle-stack-advance” (SCSA) cine stacks.

**Results:**

Bland–Altman analyses [bias (limits of agreement)] showed good agreement in left ventricular (LV) end-diastolic volume (EDV) [3.6 mL (− 5.8, 12.9)], LV end-systolic volume (ESV) [1.3 mL (− 6.0, 8.6)], LV ejection fraction (EF) [0.1% (− 4.9, 5.1)], right ventricular (RV) EDV [3.5 mL (− 3.34, 10.0)], RV ESV [− 0.23 mL (− 7.4, 6.9)], and RV EF [1.70%, (− 3.7, 7.1)] with a trend toward underestimating LV and RV EDVs with the SCSA method. Image quality was comparable for both methods (*p* = 0.37).

**Conclusions:**

LV and RV volumetric parameters agreed well between the SCSA and the conventional sequences. The SCSA method halves the breath-hold duration of the commercially available CS sequence and is a reliable alternative for volumetric analysis in a pediatric population.

**Key Points:**

*• Compressed sensing is a promising accelerated cardiovascular magnetic resonance imaging technique.*

*• We omitted the steady-state preparation cardiac cycle preceding each cine slice in compressed sensing and achieved an acquisition speed of 1 RR interval/slice.*

*• This modification called “single-cycle-stack-advance” enabled the acquisition of an entire short-axis cine stack in a single short breath hold.*

*• When tested in a pediatric patient group, the left and right ventricular volumetric parameters agreed well between the “single-cycle-stack-advance” and the conventional sequences.*

## Introduction

Left ventricular (LV) volumes and ejection fraction (EF) are the most widely used markers of cardiovascular outcome [1, 2]. They have been incorporated into clinical diagnosis and therapeutic decision-making pathways for various cardiac conditions [3, 4]. Cardiovascular magnetic resonance (CMR) is the gold standard for volumetric/functional assessment of the left and right ventricles (RV) [5–7].

The standard volumetric assessment by CMR requires acquisition of 10–12 short-axis (SAX) steady-state free-precession (SSFP) cines with retrospective triggering and separate breath holds (BHs) [8]. Conventional SAX cine stack acquisition could require up to 10 min including repeated acquisitions, especially when patients struggle with BH or the heart rate is irregular [9]. Multiple BHs are particularly difficult in children and an inconsistent expiration amplitude may cause errors by slice misalignment. Several approaches were explored previously to address this problem. “Real-time” (or “single-shot”) imaging significantly coarsens spatial and temporal resolution [10–14]. 3D imaging, often employing compressed sensing (CS) or related acceleration techniques, for ventricular volumetry overcomes interslice misalignment caused by inconsistent expiratory BH positions [15, 16]; [15, 16]; however, the entire 3D data may be degraded by respiratory motion. Real-time CS cines are promising for patients unable to tolerate long scanning sessions; several methods employing multi-slice CS real-time SSFP cines for the SAX stack were reported [17–21]. Non-Cartesian highly accelerated real-time cine imaging for the SAX stack has also been developed and applied clinically [22].

When multiple cine SSFP slices are obtained in one breath hold, SSFP “stabilization” for the next slice lasting a whole cardiac cycle is generally applied [23–25]. For real-time cine imaging, this extra cardiac cycle per slice doubles the BH time; for example, 10 slices would need a 20-cycle BH which may not be tolerated.

We applied a minor modification to the commercial multi-slice real-time CS sequence by omitting each SSFP stabilization cardiac cycle before acquiring each SAX cine slice. This modification named “single-cycle-stack-advance” (SCSA) reduced the total time required for the SAX cine stack to 1 RR interval per slice. End-diastolic measurement of each slice was obtained at the R-wave, circumventing the shortcoming that prospective cine acquisition omits late diastole. An important difference of this modification is that slice stabilization for the next slice occupies the late diastole of the previous cycle, so the real-time cine frame obtained at the R-wave has already experienced at least partial SSFP stabilization. This work aimed to investigate the feasibility of the SCSA method in a clinical setting and assessed its reliability for biventricular volume analysis in a pediatric patient population.

## Methods

### Study population

We enrolled 55 consecutive pediatric patients referred for CMR from July 2018 to March 2019. Clinical CMR protocols included both standard and SCSA SAX cine stacks. This work was prospectively registered as an audit in the Royal Brompton Hospital clinical audit register (approval number 003328), and parents provided written informed consent for teaching and research use of anonymized images. The exclusion criteria were general MRI contraindications including claustrophobia and invasively ventilated patients.

### CMR study protocol

**Fig. 1:**
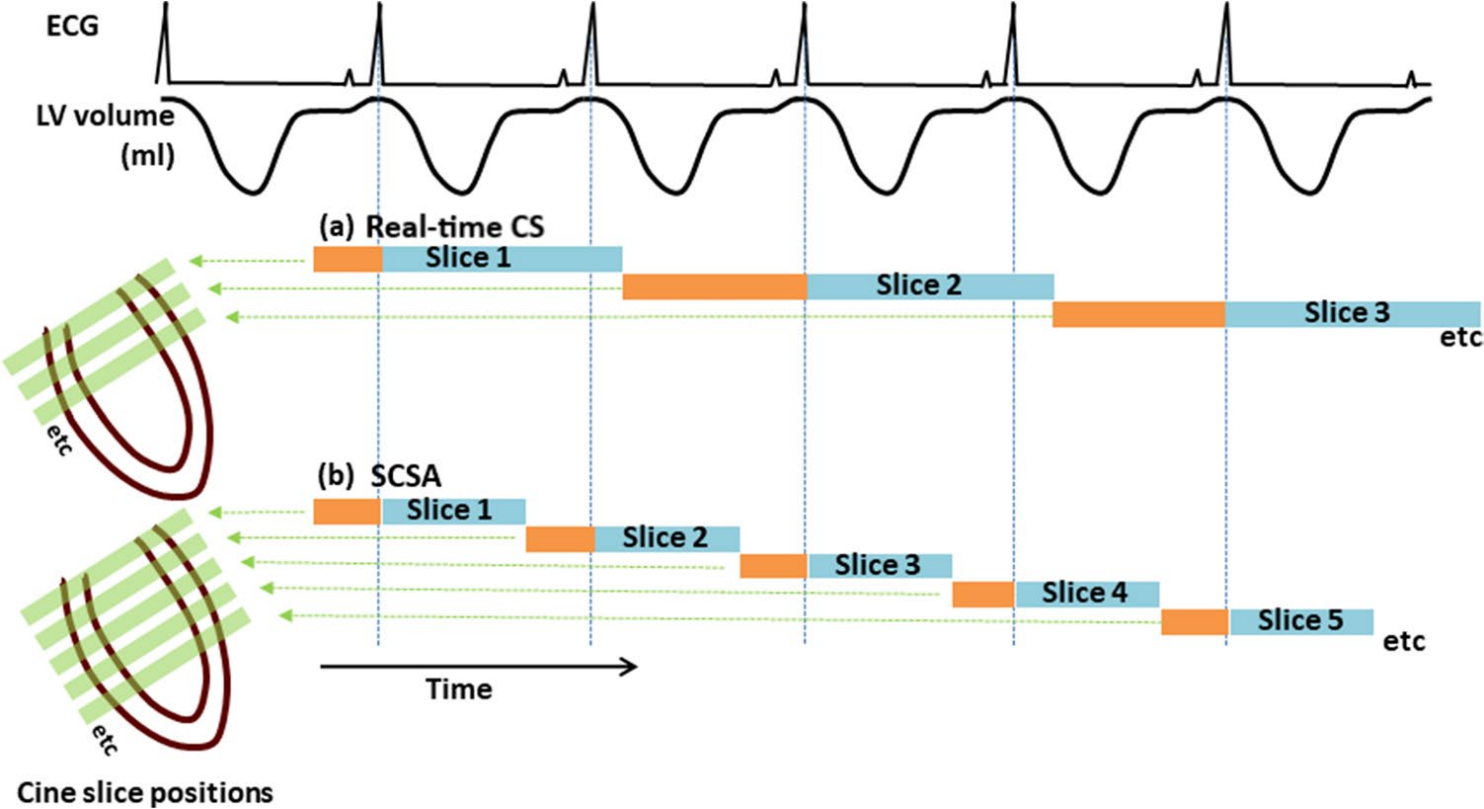
The ECG, LV volume, and cine slice positions plotted against time, showing the difference between previous real-time CS cine stack methods and the new single-cycle stack-advance method. Please note that this figure does not depict the gold-standard conventional cine applied in this work. Blue bars show cine data acquisition, and orange bars show prior SSFP stabilization of the slice. Data acquisition is inevitably slightly delayed by half the frame time after R-wave detection. (**a**) In the previous method for real-time CS for single breath-hold cine stack acquisition to capture an entire cardiac cycle, most of the following cycle is typically spent stabilizing and waiting for the next R-wave. This method was not run in this work. (**b**) The faster method evaluated in this work against conventional BH “segmented k-space” cine imaging. This modification omits late-diastolic acquisition, enabling single-cycle stack-advance (SCSA) to acquire the same number of SAX slices in half the breath-hold time. SCSA has shortened SSFP stabilization time of each slice compared to the previous real-time cine SSFP method All cines in this work were 8 mm thick with 2-mm gaps. *CS, compressed sense; SCSA, single-cycle stack-advance; SSFP, steady-state free precession*

With conventional ECG gating and array coils at 1.5 T (Magnetom Aera and Magnetom Avanto^fit^ Siemens Healthineers), the conventional SSFP SAX cine stack was acquired as for standard clinical CMR as widely described in the literature and using GRAPPA with 46 reference lines acquired during each BH [5, 7]. The SAX stack was then repeated using the SCSA modification. Unlike previous work, we aimed for the shortest BH time removing the entire unacquired cardiac cycle for SSFP stabilization of each slice [23]. Data collection captured each cardiac cycle from detection of each R-wave to early diastole, when the sequence slice location advanced automatically to the next SAX slice to deliver SSFP stabilization while waiting for the next R-wave detection. Therefore, the end-diastolic frame of each slice was obtained at each R-wave (Figure 1) having experienced some prior SSFP stabilization. This differed from conventional “prospective triggering” of the cine; because the cine sequence was already running in that slice, it was just waiting for the R-wave detection to store the data available. The conventional multiple-BH SAX cine stack parameters compared to the SCSA cine SSFP short-axis stack parameters (Table 1) were affected by body habitus in different ways; for example, a larger phase-encode FOV as a fraction of frequency-encode FOV for real-time imaging caused a slightly longer frame time, whereas for the conventional cine stack, this would usually slightly extend the BH time. An LV requiring, for example, 10 slices of SAX cines required a 10-cardiac-cycle breath hold by SCSA (Table 1). Although it is possible to perform free-breathing real-time CS imaging, a comparison of ventricular function analyses may conflate errors from respiratory misalignment. Therefore, we asked patients to comply with the short BH we achieved with the SCSA modification.

**Table 1:** Imaging parameters of the conventional SSFP cine sequence and SCSA modification of compressed sense imaging

	**Conventional SSFP**^†^	**SCSA**^#^
Cine frame time, ms	40	35–41^$$^
TR, ms	2.65	2.51
TE, ms	1.12	1.06
Field of view, mm	Typical 320–360FE × 240–280PE	Typical 320–360FE × 250–280PE (body habitus, tech mood)
Acquired spatial resolution, mm	1.7–1.9FE × 1.7–1.9PE	Nominally 1.5–1.7FE × 1.7–1.9PE
Slice thickness/gap, mm	8/2	8/2
Flip angle, °	70	70
Bandwidth, Hz/pixel	930	960
k lines/cycle^*^	15	Single-shot with CS under sampling
Other accelerations	Parallel imaging × approx 1.7	Only CS
ECG triggering	Retrospective	Prospective^##^
Breath holds, *n*	SAX slices (typical 6–10)	1
Average acquisition time	*n* * [(6–7) RR/BH + breathing time]	RR × SAX slices (typical 6–10 RR)

*ECG* electrocardiogram, *FOV* field of view, *SCSA* single-cycle stack advance; *SSFP* steady-state free precession

^†^6–10 breath holds for the same number of short-axis slices used to cover the LV in this patient population

^#^6–10 slices were acquired in a single breath hold with the speed of 1 RR interval per slice

^*^Number of k-space lines acquired per cycle into each cine image, known as segments on Siemens machines

^$$^Longer with a larger phase-encode FOV fraction of frequency-encode FOV; as with any CMR, it was helpful to minimize the phase-encode FOV across patients

^##^Prospective, but includes prior SSFP stabilization of the slice; see “Methods”

### Volumetric analysis and image quality

LV and RV volumetric analysis and LV mass measurements were performed for conventional and SCSA SAX cine stacks using cvi^42^ software (Circle Cardiovascular Imaging, version 5.6) by a single CMR experienced cardiologist (S.H., 4 years of CMR experience). Papillary muscles and LV/RV trabeculations were included in the myocardial mass calculation and excluded from the blood volume [5, 7]. Volumes were indexed to body surface area (BSA) calculated using the Mosteller formula. A random selection of 20 studies from the cohort were reanalyzed after 4 weeks and by a second experienced CMR cardiologist in order to assess intra- and interobserver variability (B.A., 3 years of CMR experience). The image quality of the standard SSFP and SCSA SAX cine stack were assessed as described in the published EuroCMR registry criteria [26]. According to these criteria, 1 point was given if an artifact impeded the visualization of more than one-third of the LV endocardial border at end systole and/or diastole on a single short-axis slice. If the artifact involved 2 or 3 slices, 2 or 3 points were given, respectively. In terms of LV coverage, 2 points were given if the apex was not covered and 3 points given if a basal slice or more than one slice in the stack was missing. An image quality score of 0 corresponded to a study with no significant artifact affecting the clinical evaluation, no missing or unusable slices, and optimal orientation of the stack. Image quality scoring was performed by an independent experienced CMR cardiologist (C.I., 10 years of CMR experience) who was not involved in the volumetric analysis.

### Statistical analysis

Continuous variables were tested by the Kolmogorov–Smirnov test for normality of distribution. Variables meeting normality were reported as mean ± SD. Median (25^th^–75^th^ percentile) values were reported for variables which were not distributed normally. Categorical data are shown in frequencies and percentages. The standard multiple-BH and the single-BH real-time SCSA techniques were compared using Bland–Altman and linear regression analyses. Variability % was calculated as the absolute difference between the two measurements divided by the mean of the two measurements. Spearman rank correlation was also applied, and the Wilcoxon signed-rank test was used to compare differences between volumetric analysis parameters obtained by two methods. Intra- and interobserver reproducibility was assessed by the interclass correlation (ICC) method. Differences in image quality scores between the standard multiple-BH and single-BH real-time SCSA technique were explored using the Wilcoxon rank-sum test. Values of *p* < 0.05 were considered statistically significant. Stata software (version 16.1, Statacorp) was used for statistical analysis.

## Results

The demographic characteristics of the study population are presented in Table 2, and the cardiac volumetry results are shown in Table 3. The median age of the 55 pediatric patients enrolled in the study was 12 years, ranging between 7 and 17 years. The study cohort consisted of 21 females and 34 males. The reasons for CMR referral were congenital heart disease in 27 patients and cardiomyopathy screening or follow-up in 28 patients. Mean heart rate during the CMR studies was 81 ± 12 beats per minute with a range 52–120. In addition to the 55 patients enrolled, a further 2 patients were omitted because of positioning errors during acquisition of the SCSA stack, and there were no other exclusions due to technical failures or extreme artifacts in the SCSA sequence. The approximate acquisition time for the conventional SSFP SAX cine stack was 7–10 min versus 6–12 s plus < 1 min of image parameters planning time for SCSA.

**Table 2:** Demographics of the study population, *n* = 55

Age, years	12 [10,11,12,13,14]^*^ years, range 7–17
Gender	21 females (38%), 34 males (62%)
BMI, kg/m^2^	19.2 ± 4.1
BSA, m^2^	1.41 ± 0.34
**Referring diagnosis**	
Cardiomyopathy screen, *n*	28
*Congenital heart disease*	
ASD/VSD/PDA, *n*	4
Repaired Fallot, *n*	6
TGA, *n*	2
Ross, *n*	2
BAV and CoA, *n*	4
Repaired DORV, *n*	1
Repaired Ebstein, *n*	1
Other valvular disease, *n*	7

*ASD* atrial septal defect, *BAV* bicuspid aortic valve, *CoA* coarctation of aorta, *DORV* double-outlet left ventricle, *PDA* patent ductus arteriosus, *VSD* ventricular septal defect

^*^Median [25^th^–75^th^ percentile]

**Table 3:** Volumetric analysis results for conventional and SCSA methods

**LV measurements**	**Conventional**	**SCSA**	**Linear regression *R***^**2**^
LV EDV, mL	105.5 [75.6–132.2]	103.5 [73.0–127.7]	0.984
LV EDVi, mL/m^2^	75.3 [62.4–84.0]	70.5 [62.8–82.2]	0.958
LV ESV, mL	34.4 [25.1–45.7]	34.1 [24.4–45.6]	0.953
LV ESVi, mL/m^2^	25.9 [20.8–31.4]	25.3 [20.7–29.6]	0.917
LV SV, mL	68.3 [52.5–80.7]	67.3 [47.4–78.8]	0.967
LV SVi, mL/m^2^	48.5 [42.1–55.2]	45.9 [41.1–52.1]	0.842
LV EF, %	64.6 [61.0–68.8]	64.4 [59.1–69.5]	0.766
LV mass, g	78.8 [60.0–99.1]	76.0 [58.5–106.0]	0.598
LV mass index, g/m^2^	55.0 [50.91–64.42]	56.2 [49.3–66.3]	0.598
**RV measurements**			
RV EDV, mL	106.6 [78.3–146.7]	100.7 [73.4–147.9]	0.994
RV EDVi, mL/m^2^	72.2 [60.0–94.5]	71.7 [57.6–90.0]	0.982
RV ESV, mL	41.0 [26.2–55.2]	40.6 [26.6–55.9]	0.957
RV ESVi, mL/m^2^	27.6 [20.3–34.3]	27.7 [20.8–35.0]	0.978
RV SV, mL	65.7 [41.0–94.0]	62.9 [35.8–86.3]	0.961
RV SVi, mL/m^2^	46.8 [38.4–56.6]	44.5 [36.1–54.5]	0.951
RV EF, %	63.5 [58.0–66.5]	61.3 [57.1–64.8]	0.797

^*^Median [25^th^–75^th^ percentile] values reported

**Fig. 2:**
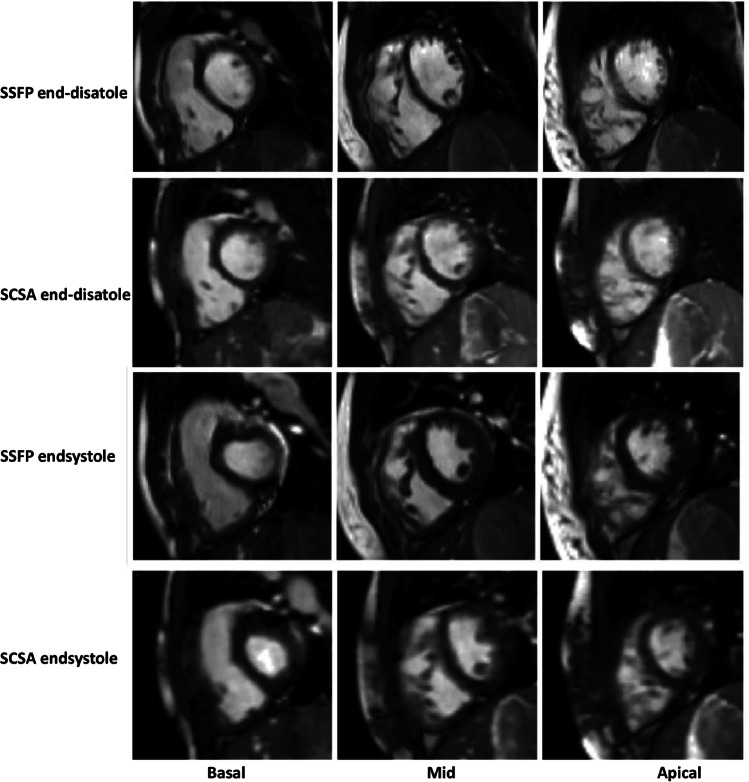
Comparative representation of corresponding (same slice location and thickness) conventional SSFP and SCSA cine images of a patient with congenital heart diease at the basal, mid-cavity, and apical levels. Although small structures such as trabeculations were adequately visualized in the CS data, the CS reconstruction may cause further degradation below the nominally similar acquired resolutions; their edges and image contrast compared to surrounding material were not as clear as in the conventional method. *CS, compressed sense; SCSA, single-cycle stack-advance; SSFP, steady-state free precession*

For the image quality scoring of the 55 patients included in the analysis, no image was given the score 2 or 3. The non-parametric paired samples Wilcoxon signed-rank-sum test showed that there was no significant difference in image quality scores (*p* = 0.37), while, interestingly, the standard SAX cine stack had more patients with score 1 than SCSA (11 vs. 8 cases, respectively). Sample images for conventional and SCSA cine stacks are shown in Fig. 2.

**Fig. 3:**
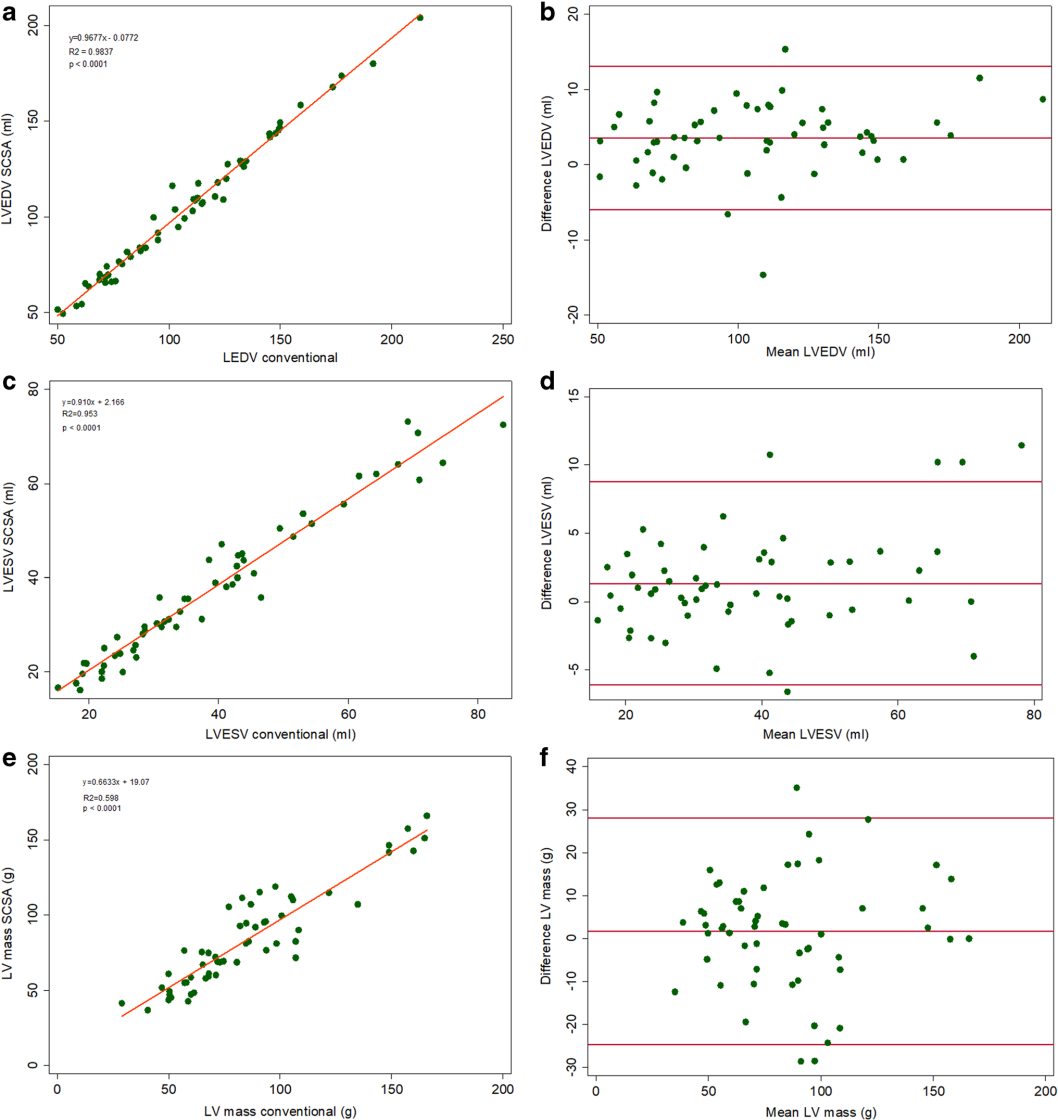
Agreement of LV parameters (**a**, **c**, **e**). Linear regression correlation (**b**, **d**, **f**), Bland–Altman. *LV, left ventricle; LVEDV, left ventricular end-diastolic volume; LVESV, left ventricular end-systolic volume*

**Fig. 4:**
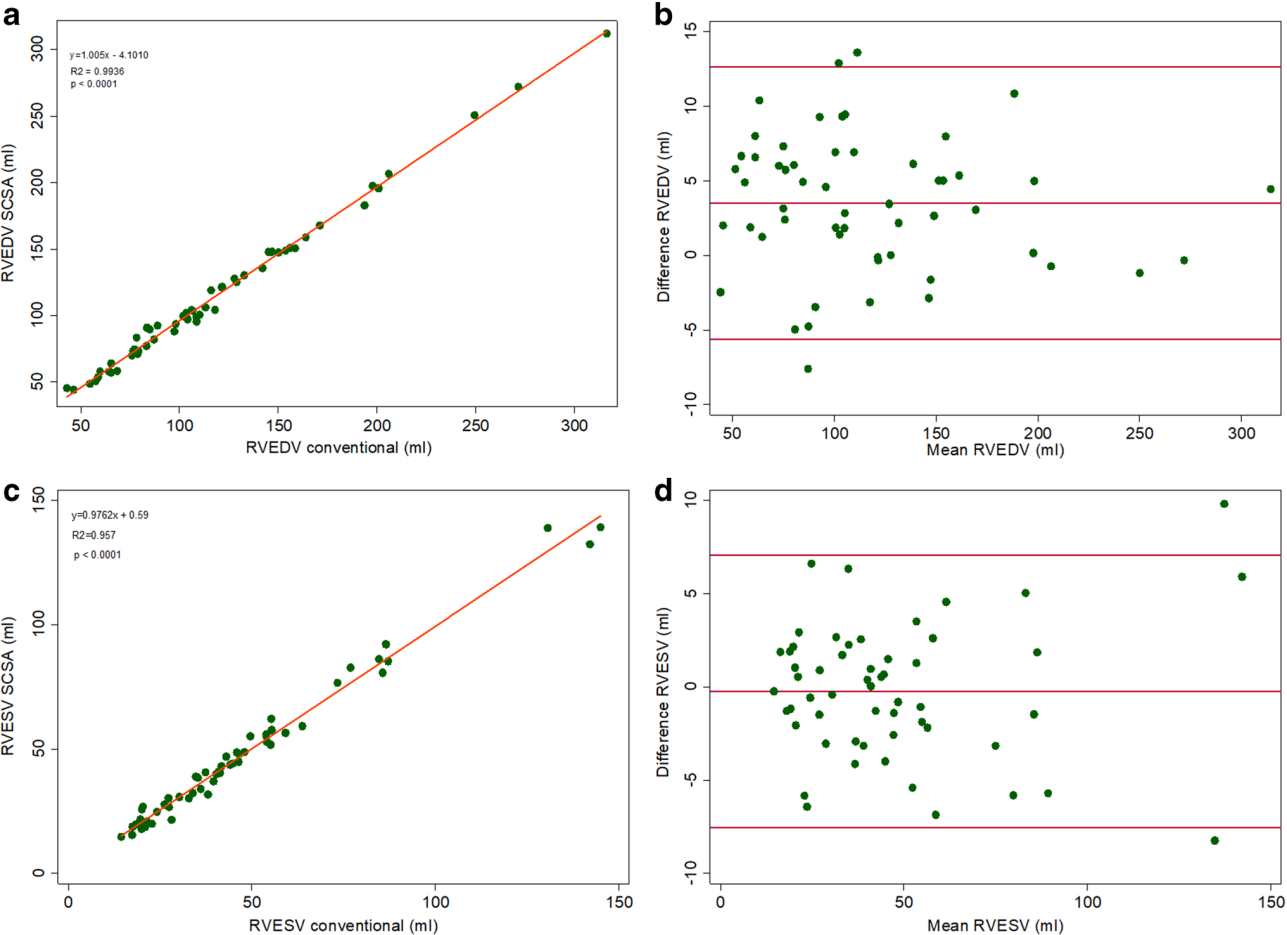
Agreement of RV parameters (**a**, **c**). Linear regression correlation (**b**, **d**), Bland–Altman. *RV, right ventricle; RVEDV, right ventricular end-diastolic volume; RVESV, right ventricular end-systolic volume*

**Fig. 5:**
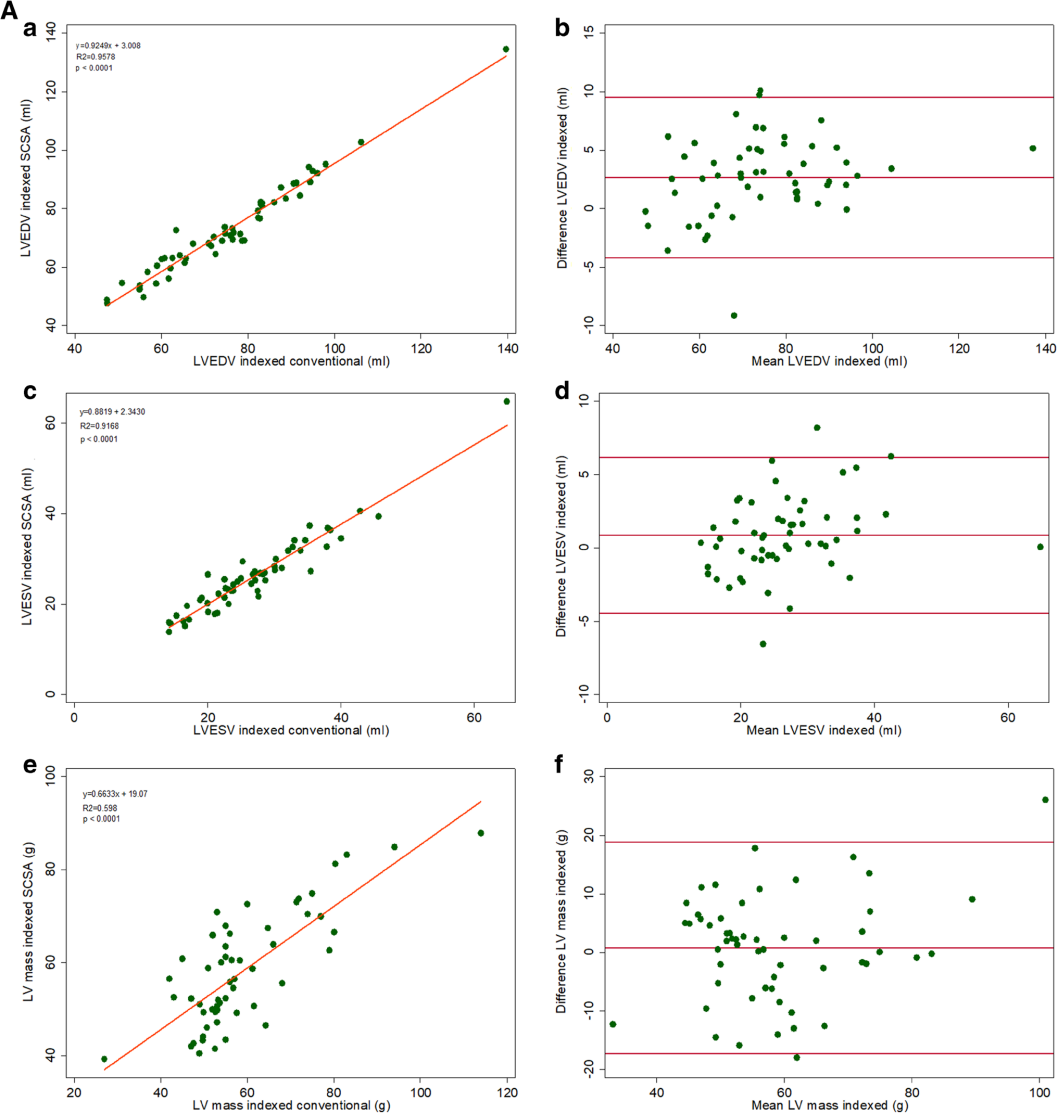

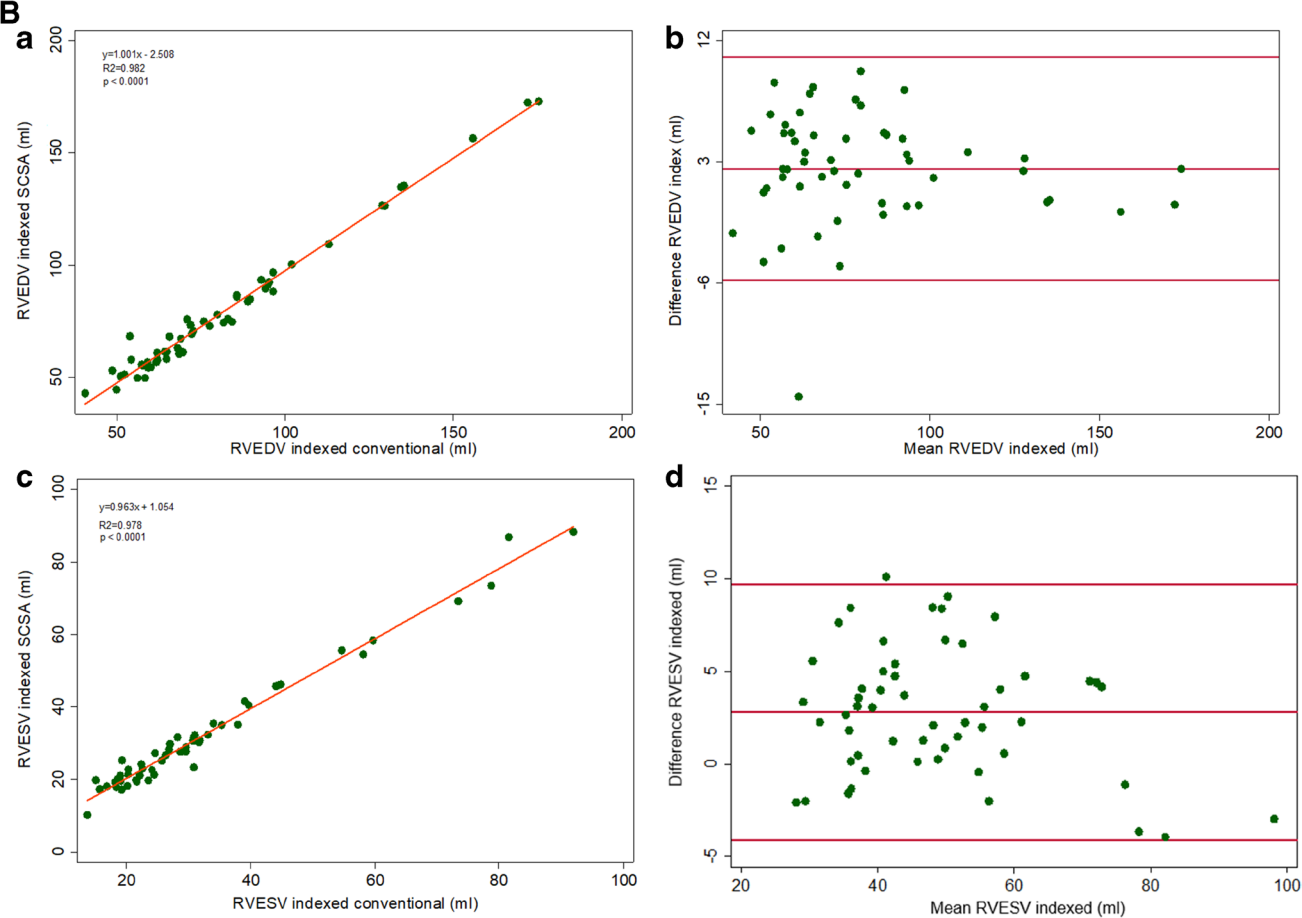
**A** Agreement of indexed LV parameters (**a**, **c**, **e**). Linear regression correlation (**b**, **d**, **f**), Bland–Altman. *LV, left ventricle; LVEDV, left ventricular end-diastolic volume; LVESV, left ventricular end-systolic volume*. **B** Agreement of indexed RV parameters (**a**, **c**). Linear regression correlation (**b**, **d**), Bland–Altman. *RV, right ventricle; RVEDV, right ventricular end-diastolic volume; RVESV, right ventricular end-systolic volume*

The conventional SAX cine acquisitions were used as the “gold standard” for the measurement of LV end-diastolic volume (EDV), LV end-systolic volume (ESV), LV EF, LV mass, RV EDV, RV ESV, and RV EF. LV EDV and RV EDV had good agreement but were systematically underestimated by the SCSA method (Figures 3 and 4). The limits of agreement and variability were in the clinically acceptable range (Table 4). Contrary to the LV ESV which followed the same trend of underestimation by the SCSA method (variability 3.1 ± 9.4%, *p* = 0.010), the RV ESV had a trend to be overestimated (variability − 0.1% ± 6.4, *p* = 0.646). When the RV and LV volumes were indexed to the BSA, closer agreement was achieved between SCSA and conventional methods (Figure 5a–b).

**Table 4:** Differences in ventricular volumetric parameters between conventional and SCSA methods

	**Difference** **(conventional − SCSA)** **(mean ± SD)**	**Variability (%)** **(mean ± SD)**	p^*^	**Correlation**^†^
LVEDV	3.6 ± 4.8 mL	3.5 ± 4.9	< 0.001	*r* = 0.985, *p* < 0.001
LVESV	1.3 ± 3.7 mL	3.1 ± 9.4	0.010	*r* = 0.973, *p* < 0.001
LVEF	0.1 ± 2.5%	0.1 ± 4.0	0.759	*r* = 0.848, *p* < 0.001
LV Mass	1.7 ± 13.2 g	2.2 ± 16.1	0.349	*r* = 0.890, *p* < 0.001
RVEDV	3.5 ± 4.6 mL	3.8 ± 5.3	< 0.001	*r* = 0.993, *p* < 0.001
RVESV	− 0.2 ± 3.7 mL	− 0.1 ± 6.4	0.646	*r* = 0.985, *p* < 0.001
RVEF	1.7 ± 2.8%	2.7 ± 4.5	< 0.001	*r* = 0.867, *p* < 0.001

Bland–Altman plots highlight the mean difference and the standard deviation of the difference between the two measurements. Variability [%] is the absolute value of the difference between the two measurements divided by the mean of the two measurements

^*^The Wilcoxon signed-rank test was used to compare the paired difference in volumetric analysis parameters between conventional and SCSA method measurements

^†^Spearman rank correlation

**Fig. 6:**
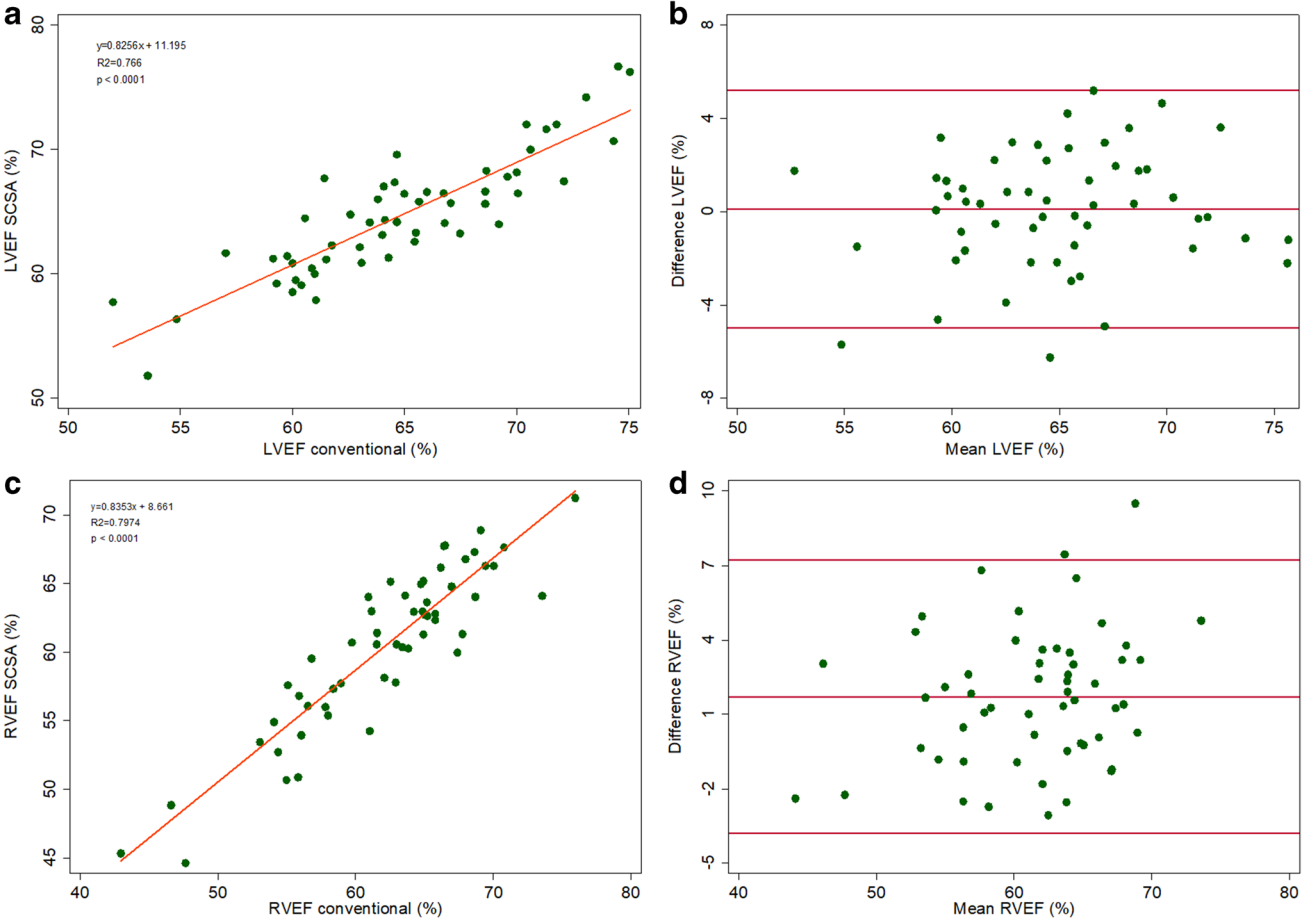
Agreement between LV and RV EF (**a**, **c**). Linear regression correlation (**b**, **d**), Bland–Altman. *LV, left ventricle; RV, right ventricle; EF, ejection fraction*

The LV EF and RV EF by SCSA showed close agreement with the gold standard (Figure 6). The difference between the gold standard and SCSA in the LVEF values was not statistically significant and for the RVEF [bias 1.70% (limits of agreement − 3.7, 7.1)], and there was a systematic underestimation by SCSA, but variance was within clinically accepted limits (Table 4).

The LV mass and BSA-indexed LV mass calculated with the two image acquisition methods had moderate agreement (Figures 3 and 5A). Table 5 shows the inter- and intraobserver variability expressed as ICCs, where it can be seen that there is no significant variability in the readings between the conventional and SCSA acquisition methods.

**Table 5:** Inter- and intraobserver agreements of readings obtained from conventional and SCSA SAX cine stacks (*n* = 20)

Interobserver			Intraobserver	
Variable	ICC Conv	ICC SCSA	ICC Conv	ICC SCSA
LVEDV	0.987	0.992	0.996	0.995
LVESV	0.969	0.930	0.977	0.978
LVEF	0.861	0.837	0.891	0.774
LV Mass	0.943	0.919	0.953	0.945
RVEDV	0.994	0.980	0.997	0.995
RVESV	0.981	0.993	0.991	0.983
RVEF	0.861	0.814	0.820	0.765

## Discussion

In the current study, we describe the clinical evaluation of a minor modification to the real-time (“single-shot”) CS SSFP multi-slice cine sequence (SCSA), which accelerates the SAX stack acquisition speed to one R–R interval for each required slice, thus halving the breath-hold time compared to the use of steady-state preparation cardiac cycles incorporated in most of the previous real-time CS cine methods. The SCSA modification was tested during clinical pediatric CMR scans and proved to be reliable in terms of RV/LV volumetric analysis compared to the gold standard of multiple-BH SSFP SAX cine stack. It was also proved practical to apply this method in a busy clinical setting, because no pre-scans or sequence adjustments were necessary, except the usual good practice of minimizing the phase-encode FOV along the shortest available direction across the patient. To the best of our knowledge, this is the first clinical report describing/assessing the clinical applicability and reliability of a real-time CS cine SAX stack sequence with an overall acquisition speed of a single RR interval per slice.

The agreement for the LV EF values was good with no significant difference between the two acquisition methods, whereas LV EDV, LV ESV, RV EDV, and RV EF were systematically underestimated by the SCSA method, but the differences for all the parameters (Table 4) were comparable to the interobserver variability reported in the literature for conventional cine SSFP [27–30]. The RV ESV was the only parameter with a tendency to be overestimated by the SCSA method, but the difference was not statistically significant. Bland–Altman and linear regression analyses yielded good agreement for all six volumetric parameters. Our patient population included cases with ventricular dilation and mild RV/LV dysfunction, in which satisfactory agreement results for biventricular volumes and EF with both methods proves that there was no difference in identifying presence of dilation or ventricular dysfunction. The LV mass and LV mass index were also underestimated by SCSA and showed moderate agreement between the two acquisition methods.

The omission of late-diastolic imaging by the SCSA acquisitions could cause such an underestimation of the EDVs if, for example, the increase in LV volume during atrial contraction were missed [19, 21, 24]. However, in the SCSA sequence, the image obtained at the R-wave was not subject to any SSFP stabilization delays (because stabilization was performed during the previous late diastole; see Figure 1) and this image can reasonably be regarded as showing the truly maximal ventricular volume. Alternatively, with the image degradation associated with the application of CS especially of small features, the endocardial border, and consequently the blood-filled intertrabecular spaces, might be less well detected, potentially resulting in a “smaller” endocardial contour and underestimation of EDVs. The compacted nature of the LV myocardium in systole and loss of further trabeculations from the cavity might have also caused the same trend for LV ESV. The RV trabecular pattern is usually more extensive than in the LV, and it is also probable that small features such as intracavitary trabeculations were not depicted correctly by the real-time CS cine; as our analysis policy would exclude noted RV trabeculations from the RV ESV, the overestimation trend observed in our measurements from the images might be a consequence of this fact. Some unquantifiable degradation of the achieved spatial resolution in the CS method might have interfered with epicardial and endocardial border detection resulting in moderate agreement for LV mass; however, variability of the LV mass parameter is also reported to be high with the conventional multiple-BH cine stack method [26, 29]. Additionally, we have also reported the BSA-indexed cardiac volumetry values and, as would be expected from this pediatric sample, the graphs showed enhanced agreement when the BSA-indexed values were plotted.

Interestingly, the standard multiple-BH SAX cine stack had more patients with score 1 than SCSA. This may have resulted from the innate robustness of real-time imaging to poor breath-holding. Also, the criteria used by the image quality scoring were based on the images’ usability for volumetry analysis and do not reflect their utility for more subtle aspects.

There are previous reports on CS applications assessing ventricular function. Although the image quality of each real-time image at short frame times is considered robust to respiratory motion [16], for clinical application, the respiratory misregistration of the heart between the slices of the SAX stack is a drawback. Therefore, it is important to be clear that this drawback still drives a clinical requirement to obtain the SAX cine stack during a single BH. Even though the imaging method itself is not degraded by free-breathing application, the accuracy of ventricular volume analysis over the whole stack is degraded. This loss of accuracy is partially caused by through-plane mislocation of the heart, and it is not fully corrected simply by aligning the heart “in-plane” from one cine slice to the next. The recent report by Kocaoglu et al evaluating BH and free-breathing CS for quantitative assessment of biventricular volumes in 26 children and young adults (aged between 9 and 35 years) with cardiomyopathy showed that volumetric analysis for free-breathing CS acquisition was comparable (although with a statistically significant difference from two BH methods applied) to conventional BH acquisition with SENSE factor 2 but scan time was longer, blood to myocardial contrast was lower, and image quality was compromised (good to adequate vs. excellent to good) with the free-breathing method [31]. Free-breathing temporal resolution was assessed in 26 subjects, including a real-time CS cine stack acquired in 15 cases at 39 ms, and resulted in larger errors compared to the conventional method potentially arising from misregistration [20].

Persistent underestimation of LV EDV in previous CS volumetry reports listed is consistent with our findings. Vermersch et al employed multi-slice 2D CS real-time SSFP SAX stack cines in comparison to the conventional method, in 100 consecutive patients over a wide range of cardiac function, requiring two cardiac cycles (i.e., stabilization and imaging) per cine slice. The diagnostic performance compared well against conventional BH cines, including for the detection of wall-motion disorders and regurgitant valve flow, finding only a small but statistically significant underestimation of LV EDV (− 1.8%) and overestimation of LV mass (+ 1.9%) by the real-time cine CS. There was also an underestimation trend for RVEDV similar to our work [21]. The underestimation of LV EDV was also reported using real-time CS in a different approach, obtaining 7 cines (mixed long- and short-axis method) in 14 cardiac cycles [19]. Notably, in common for all these previous works, a cardiac cycle of SSFP stabilization was required before proceeding with each SAX cine slice. By omitting the stabilization cycle of each slice, we achieved a total acquisition speed of a single RR interval per SAX cine slice.

### Limitations

Real-time CS imaging may be adequate for biventricular EF and volume measurements, whereas the evaluation of small pathological structures as present in cardiomyopathies or congenital heart diseases is generally considered to be more reliable when performed on conventional cine images, if supported by breath-holding and reasonably normal cardiac rhythm. As with any CS or accelerated method, coarser spatial and temporal resolution than the stated nominal resolution may occur. Also, the sequence was prospectively triggered and although we believe that it captured the maximal ventricular volume at the R-wave as explained above, it did not capture other cardiac motions during the late-diastolic phases of the cardiac cycle which may be important in certain other clinical questions. The time constraints of a busy clinical service prevented adding another scan to test the free-breathing versions of CS. Another limitation potentially occurs if the preceding cycle is short, so that the time for SSFP stabilization in the next slice is short (in the region of 50 ms) before the R-wave occurs and data acquisition is enabled. In this situation, the image contrast between the blood and myocardium will not have developed fully, because this arises from the myocardial T2 and may require in the region of 80–100 ms to fully darken the myocardium in the SSFP sequence. However, this effect was not observed, nor were any SSFP stabilization artifacts, because the SCSA acquisition window was set to ensure that stabilization took place for long enough before data acquisition began. The reconstruction times on two unmodified clinical CMR systems (with GPU upgrades as sold by the manufacturer) were unobtrusive and did not prevent the following scan from proceeding.

## Conclusion

SCSA yielded comparable quantitative volumetric results as conventional SAX cine stack with several BHs in a pediatric cohort referred for assessment of congenital heart disease or cardiomyopathy.

## Abbreviations

BH: Breath hold
BSA: Body surface area
CMR: Cardiovascular magnetic resonance
CS: Compressed sense
ECG: Electrocardiography
EDV: End-diastolic volume
EF: Ejection fraction
ESV: End-systolic volume
FOV: Field of view
LV: Left ventricle
RV: Right ventricle
SAX: Short axis
SCSA: Single-cycle-stack-advance
SSFP: Steady-state free precession

## Declarations

### Guarantor

The scientific guarantor of this publication is Peter Gatehouse, Senior Clinical CMR Physicist, Royal Brompton Hospital.

### Statistics and biometry

Author Winston Banya has significant statistical expertise. He is a statistician employed by Research Office, Royal Brompton & Harefield NHS Foundation Trust, London, UK.

### Study subjects or cohorts overlap

Some study subjects have been previously reported in abstract presentations in ISMRM 27^th^ Annual Meeting 11–16^th^ May, Montreal, QC, Canada, and SCMR 22^nd^ Annual Scientific Sessions 6–9^th^ February 2019.

### Methodology

• prospective

• cross-sectional study

• performed at one institution
